# Complete genome of multiply antibiotic resistant ST10 *Acinetobacter baumannii* isolate NL6 from Vietnam and relationship to available ST10 genomes

**DOI:** 10.1128/spectrum.01926-26

**Published:** 2026-09-09

**Authors:** Stephanie J. Ambrose, Ruth M. Hall

**Affiliations:** 1 School of Life and Environmental Sciences, The University of Sydney 164115, Sydney, NSW, Australia; 2 Sydney Infectious Diseases Institute, The University of Sydney 164115, Sydney, NSW, Australia; Griffith University - Gold Coast Campus, Gold Coast, Australia

**Keywords:** *Acinetobacter baumannii*, ST10, resistance plasmids, integrative element, ISAba1, ISAba26

## Abstract

The genome of NL6, a multiply antibiotic-resistant *Acinetobacter baumannii* ST10:KL49:OCL2 carriage isolate from Vietnam, was sequenced using Nanopore technology, and complete chromosome and plasmid sequences were assembled from the long reads and available short reads. Resistance genes and their locations were identified, and transfer of a conjugative plasmid carrying several resistance genes into a new host was tested. The acquired resistance genes in NL6 were distributed between the chromosome and two of three plasmids present. The chromosome carries multiple copies of several insertion sequences, an incomplete copy of the ISAba1-bounded Tn*6250* that includes the *sul2* and *strAB* genes, and an integrative element carrying copper resistance genes designated IECu^R^. Plasmid pNL6-2 (r3-T5; 15 Kbp) is a Rep_3/OrfX plasmid that includes a *tet39 dif* module, and pNL6-3 (r3-T20; 66.9 Kbp) carries *aacC2d*, *aphA6*, and *bla*_CARB-16_ and a second *ampC* gene preceded by an ISAba1. Conjugation of pNL6-3 into derivatives of ATCC17978 was demonstrated, confirming that the *ampC* gene confers resistance to third-generation cephalosporins. NL6 was compared to other complete ST10 genomes. Several acquired elements in the chromosome were shared with the ST10 isolate LAC-4 (USA), indicating shared ancestry, but the plasmid content differed. The KL and plasmid content were variable in 17 further complete ST10 genomes downloaded from GenBank. Tn*6250* and IECu^R^ were only found together in the chromosome of two further KL49 isolates. Antibiotic resistance in ST10 *A. baumannii* was acquired mainly via plasmid acquisition, but resistance genes varied, and a variety of plasmids was involved.

**IMPORTANCE:**

Members of the CC10 clonal complex of *Acinetobacter baumannii* comprising ST10 plus single and double locus variants are known to be particularly virulent. However, antibiotic resistance in members of this group has rarely been examined. Here, determination of the complete genome (chromosome and plasmids) of a representative ST10 isolate from Vietnam allowed the context and location of acquired antibiotic resistance genes and of other mobile genetic elements to be determined. Mobile genetic element locations in completed chromosomes facilitate comparisons of potentially related genomes, revealing those with recent shared ancestry. Differences in plasmid content can also be examined.

## INTRODUCTION

The context of antibiotic resistance genes in *A. baumannii* globally disseminated clones GC1 and GC2 is well characterized, and they are mainly found in complex resistance regions in the chromosome (1–8), though plasmids also contribute to the dissemination of antibiotic resistance genes (9–14). However, the context of antibiotic resistance genes in non-GC1, non-GC2 isolates is not as well understood, with less attention paid to how these isolates have become multiply or extensively antibiotic resistant.

ST10 *A. baumannii* is a clone of increasing importance, and ST10 carrying the KL49 capsule biosynthesis locus has been reported to be virulent. This is evidenced by high mortality rates in a hospital outbreak among immunocompetent patients in the USA (15), increased serum resistance (16, 17), and observed hypervirulence (and increased mortality) in both mouse (15, 18) and *Galleria* (17, 19) models of infection using LAC-4 and other ST10 strains. LAC-4 was isolated in the USA in 1997 (20) and has widely been used in experimental studies (18, 21–23). A complete genome for LAC-4 was reported in 2015 (24).

ST10 isolates have been reported from clinical sources, including hospital-acquired infections and outbreaks (15, 20, 25–32), community-acquired infections (19, 33), and asymptomatic carriage isolates (28) from a range of countries. Indeed, ST10 was the most common ST (>50% of isolates) identified in a study of community-acquired *A. baumannii* infections in the Northern Territory of Australia, although all were antibiotic susceptible (33). It was also the most common type found during an investigation of hospital-acquired *A. baumannii* in patients from a hospital in Iran (27). ST10 has also been found in samples from animals (34, 35) and the environment (36).

ST10 isolates were found among both carriage and infection isolates at the Hospital for Tropical Diseases, Ho Chi Minh City, Vietnam (28). NL6 is an ST10:KL49:OCL2 (Oxford scheme ST447) *A. baumannii* carriage isolate recovered in 2003. Recently, it was used to determine the structure of the K49 capsular polysaccharide (37). Here, we show that NL6 is a multiply antibiotic-resistant isolate and determine the complete genome to build a detailed picture of the resistance regions. The complete genome of NL6 was also compared with that of LAC-4 and other complete ST10 *A. baumannii* genomes available in the GenBank non-redundant database.

## RESULTS AND DISCUSSION

### Complete genome of NL6

**Fig 1:**
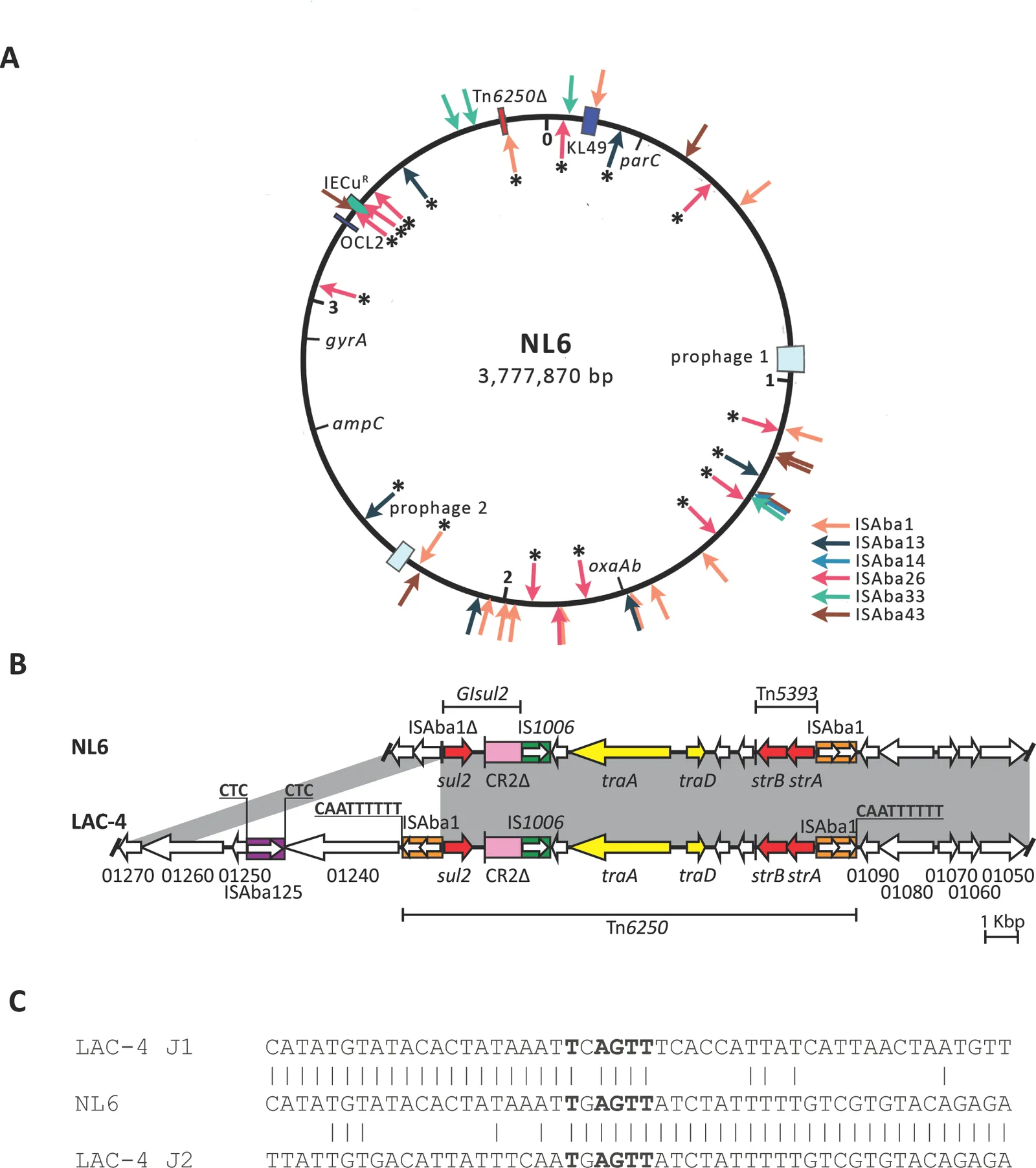
Transposons and insertion sequences in the NL6 chromosome. (**A**) Circular map of the NL6 chromosome. Numbers represent the chromosome position in megabases, with 0 indicating the origin of replication. A red box indicates the position of transposon Tn*6250*Δ. Dark blue boxes indicate the capsule locus (KL49) and the outer core locus (OCL2). Light blue boxes indicate putative prophage. The position of some chromosomal genes is shown. Insertion sequence (IS) positions are indicated by arrows and colored according to the key. IS positions shared between NL6 and LAC-4 are indicated by a * above the arrow. Drawn to scale from GenBank accession number CM146746. (**B**) Structure of Tn*6250*Δ in NL6 and comparison to Tn*6250* in LAC-4. The extent and orientation of genes and open reading frames are indicated by arrows. Antibiotic resistance genes are red; mobilization genes are yellow; and all other open reading frames are white. IS are indicated by colored boxes; ISAba1 is orange; IS*1006* is green; and ISAba125 is purple, with an internal arrow indicating the orientation of the transposase gene. CR2 is pink, with the *ori* end indicated by a vertical black bar. IRs are shown as vertical bars. The extent of Tn*6250* and regions derived from GI*sul2* or Tn*5393* are indicated. Drawn to scale from GenBank accession numbers CM146746 (NL6) and CP007712 (LAC-4). Gray shading indicates regions shared between LAC-4 and NL6. (**C**) Alignment of the left Tn*6250*Δ junction with the chromosome in NL6 (bases 3,662,997 to 3,663,046 from CM146746) with a region of the chromosome in LAC-4 (LAC-4 J1; bases 140,338 to 140,387 of GenBank accession CP007712) and the left Tn*6250* junction (LAC-4 J2; bases 132,493 to 132,542 from CP007712). Shared bases are indicated by a vertical line. The sequences for LAC-4 J1 and LAC-4 J2 were reverse complemented for ease of comparison.

The complete genome of NL6 (ST10:KL49:OCL2) was determined by hybrid assembly of Nanopore long-read data and Illumina MiSeq short-read data. The genome consists of a 3.78 Mbp chromosome and three plasmids pNL6-1 (2,471 bp), pNL6-2 (15,038 bp), and pNL6-3 (66,913 bp) (Table 1). The intrinsic *oxaAb* gene in the chromosome (which does not contribute to resistance) encodes the OXA-68 variant, which was also seen in LAC-4 and other ST10 isolates. A total of 43 IS were found in the chromosome of NL6, and their locations are shown in Fig. 1A. A total of 12 complete copies of ISAba1 were present in the chromosome, including the copy that interrupts *itrB2* in KL49 as described previously (37). However, an ISAba1 was not present upstream of the *ampC* gene. Copies of ISAba13 (*n* = 7), ISAba14 (*n* = 1), ISAba26 (*n* = 12), ISAba33 (*n* = 4), and tISAba43 (*n* = 6) were also found. Several of these IS are present in the same position in the reference ST10 strain LAC-4 (marked in Fig. 1A and see below), consistent with shared ancestry. Two putative prophage regions were detected in the chromosome using PHASTEST (Fig. 1A).

**TABLE 1:** Properties of the NL6 chromosome and plasmids

	Size (bp)	Plasmid type^*a*^	Copy number^*b*^	Resistance genes	Accession
Chromosome	3,777,870	–^*c*^	*–^c^*	*sul2*, *strAB*	CM146746
pNL6-1	2,471	r3-T204^*d*^	29.11	–^*e*^	JBPCCI020000002
pNL6-2	15,038	r3-T5	2.99	*tet39*	JBPCCI020000003
pNL6-3	66,913	r3-T20	0.98	*ampC*, *bla*_CARB-16_, *aacC2d*, *aphA6*	JBPCCI020000004

^*a*^ Rep type determined using the *Acinetobacter* plasmid typing tool (38).

^*b*^ The copy number was determined from the Illumina read depth for the plasmid relative to the average read depth of the seven MLST^IP ^alleles.

^*c*^ Not applicable.

^*d*^ Novel Rep type identified here.

^*e*^ No antibiotic resistance genes are present.

### Antibiotic resistance in NL6

NL6 was found to be multiply antibiotic resistant but susceptible to carbapenems (imipenem, meropenem, doripenem) and ampicillin/sulbactam. It was resistant to ampicillin, ceftazidime, cefotaxime, ceftriaxone, cefepime, and piperacillin-tazobactam, nalidixic acid, ciprofloxacin, and levofloxacin, trimethoprim, sulphamethoxazole, streptomycin, tetracycline, and doxycycline, gentamicin, tobramycin, kanamycin, neomycin, and amikacin. It was susceptible to spectinomycin, netilmicin, minocycline, and rifampicin.

We examined the causes of antibiotic resistance and found that several acquired antibiotic resistance genes are distributed between the chromosome and two of the plasmids (pNL6-2 and pNL6-3) (Table 1). The fluoroquinolone resistance can be explained by known mutations in *gyrA* and *parC* leading to the single amino acid substitutions in GyrA (S81L) and ParC (S84L). Although none of the ISAba1 were upstream of *ampC* or *oxaAb* in the chromosome, third-generation cephalosporin resistance can be explained by a second *ampC* copy found in pNL6-3 with an ISAba1 9 bp upstream in the correct orientation to supply the internal ISAba1 promoter. The 3,656 bp *ampC* segment located between the ISAba1 and a copy of ISAba33 (ISAba1 and ISAba33 share 88.5% DNA identity) is chromosomally derived but shares only 98.39% identity with the equivalent region in the NL6 chromosome. Searches in the GenBank non-redundant database identified four *A. baumannii* chromosomes, AB105 (ST2266; CP103338), A207 (ST152; CP132915), Ab-C102 (ST1472; CP051862), and 144AB_F11_FAQ07850_BC02 (ST1405; CP196888), with >99.9% DNA identity across this region. Though none of these isolates had ISAba1 upstream of *ampC*, this segment was likely mobilized from a close relative of one of them.

An ISAba1 copy was associated with the *strA* and *strB* streptomycin resistance determinants and *sul2* sulphonamide resistance gene (Fig. 1B). This region is part of the ISAba1-bounded transposon Tn*6250* that was first reported in the chromosome of LAC-4 (24). In NL6, it is in the same location as Tn*6250* in LAC-4 (Fig. 1B), but there has been a deletion extending from within the ISAba1 at the *sul2* end of Tn*6250* to a position in the chromosome 6,694 bp away. This deletion leaves only 29 bp of the ISAba1 and appears to have arisen by recombination between two very short regions of homology to form Tn*6250*Δ (Fig. 1C).

The *tet39* tetracycline resistance determinant, which confers resistance to tetracycline and doxycycline but not minocycline (39, 40), is present in pNL6-2 and is part of a *dif* module. Three further resistance genes were detected in pNL6-3, *bla*_CARB-16_, *aphA6* (confers resistance to amikacin, kanamycin, and neomycin), and *aacC2d* (confers resistance to gentamicin and tobramycin). A trimethoprim resistance gene was not found, and the *folA* gene was identical to that in MSHR_A204 (33), a trimethoprim-sensitive ST10 isolate. Acquired antibiotic resistance genes or alterations affecting chromosomal genes thus accounted for all the observed antibiotic resistance phenotypes, except trimethoprim resistance.

### pNL6-3, a conjugative plasmid carrying multiple antibiotic resistance genes

**Fig 2:**
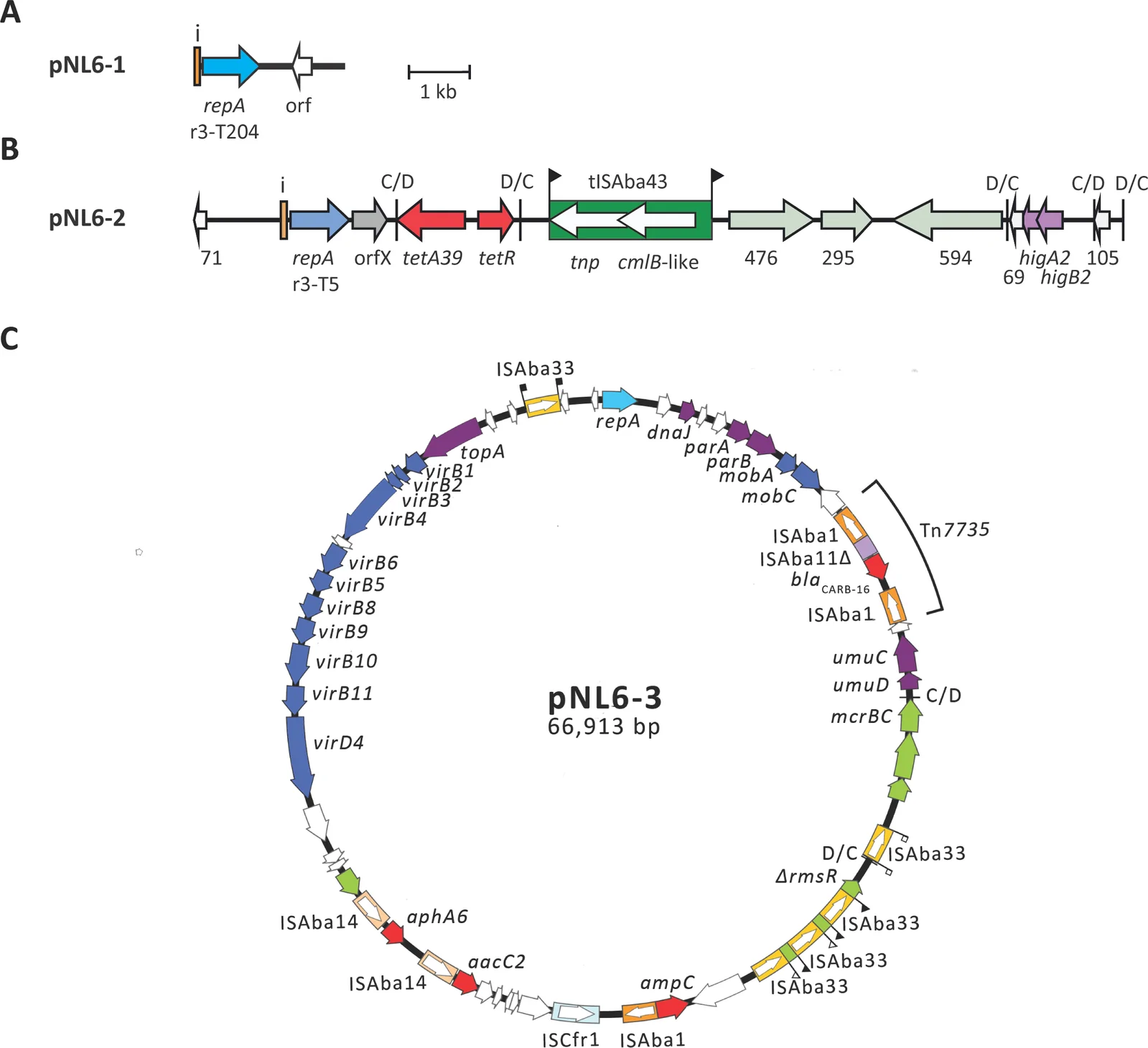
Structure of plasmids in NL6. (**A**) pNL6-1, (**B**) pNL6-2, and (**C**) circular map of pNL6-3. Arrows indicate the extent and orientation of genes and open reading frames. *repA* genes are light blue; antibiotic resistance genes are red; genes involved in DNA metabolism are purple; genes involved in conjugative transfer are dark blue; genes associated with restriction modification systems are green; and other open reading frames are white. The location of the iterons is indicated by an orange box. Insertion sequences are shown as colored boxes with an arrow inside to indicate the orientation of the transposase gene; ISAba1 is orange; ISAba33 is yellow; ISAba14 is pale orange; ISCfr1 is light blue; and tISAba43 is green. Target site duplications are indicated by open or closed triangular, square, or circular flags. p*dif* sites are indicated by a vertical bar, with the orientation of the site (C/D or D/C) above. In (**C**), the extent of Tn*7735* is indicated. Figures are drawn to scale from GenBank accession numbers listed in Table 1.

The largest plasmid, pNL6-3 (66,913 bp), has a copy number approximately equal to that of the chromosome (Table 1). It is an r3-T20 type plasmid, as the *repA* gene in pNL6-3 shares 99.67% DNA identity with the *repA* gene of pRCH51-3 (KY216144; [41]), the reference r3-T20 plasmid in the *Acinetobacter* plasmid typing database (38). A map showing the location of *ampC* and the three other antibiotic resistance genes it carries (*bla*_CARB-16_, *aphA6*, and *aacC2d*) is shown in Fig. 2C. There is no target site duplication (TSD) around the ISAba1 and ISAba33 bounded region that includes *ampC* (described above), and although a transposon carrying the *oxa23* gene with an ISAba1 at one end and ISAba33 at the other end has been described (28, 42), the exact method of mobilization cannot be determined. Of note, the adjacent gene is incomplete, with only the last 912 bp of the 2,628 bp gene present, indicating that there has been a deletion. There has also been a duplication of 365 bp of the gene between copies of ISAba33. A copy of ISAba33 with a 9 bp TSD has also interrupted an open reading frame near the *repA* gene (Fig. 2C).

The *bla*_CARB-16_ β-lactamase gene in pNL6-3 is in a transposon, Tn*7735*, which is bounded by directly oriented copies of ISAba1 and first described in the chromosome of several ST164 *A. baumannii* strains from China (43). Tn*7735* interrupts a gene encoding a hypothetical protein and has generated a 9 bp TSD (ATTCTTTCT). It includes a partial copy of ISAba11 upstream of *bla*_CARB-16_ (Fig. 2C). The *aphA6* and *aacC2d* genes are in close proximity and both preceded by a copy of ISAba14, but evidence for a larger mobile element was not found.

**Fig 3:**
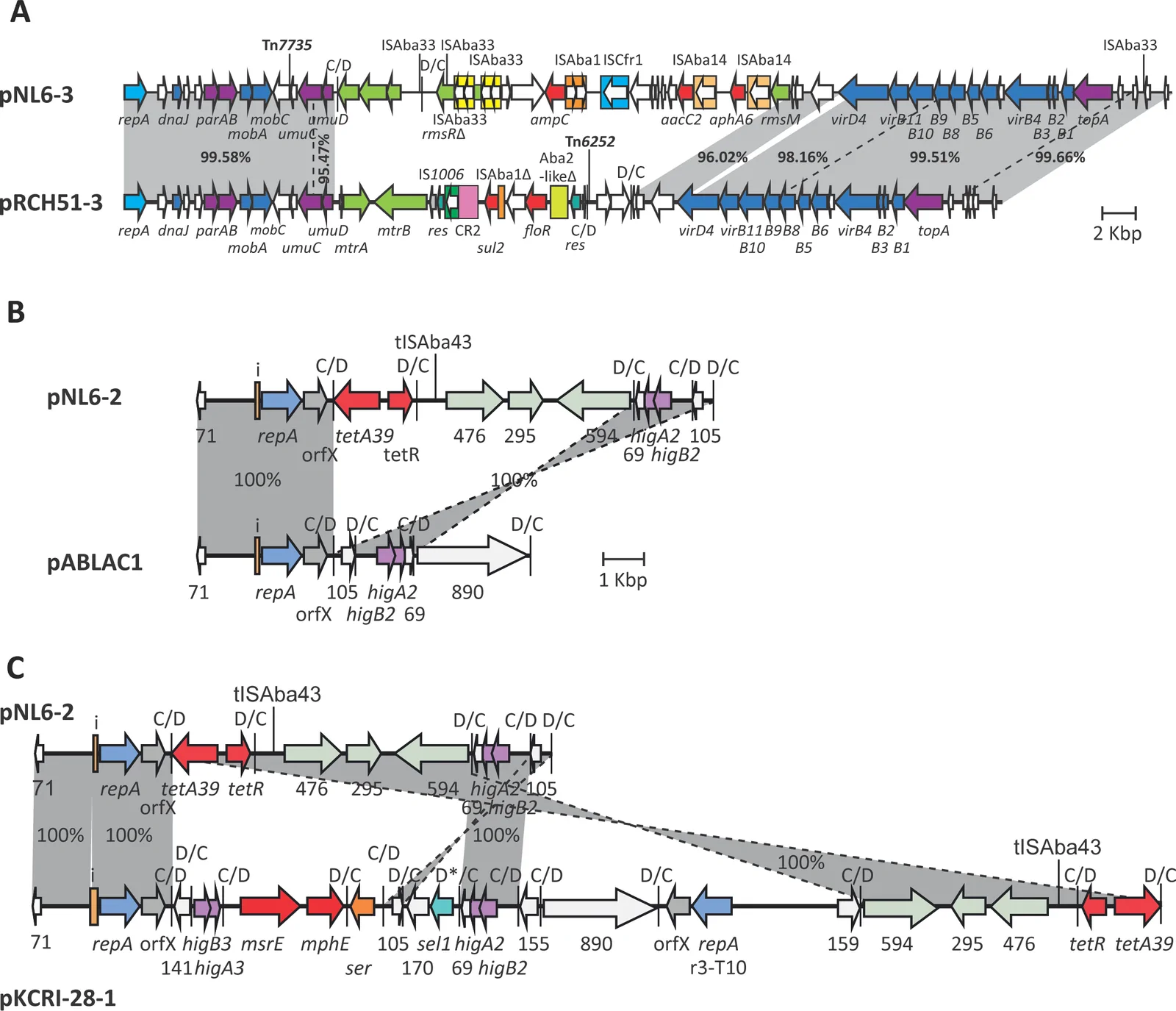
Relationship of plasmids in NL6 with available plasmids in GenBank. (**A**) Comparison of pNL6-3 with pRCH51-3. Comparison of pNL6-2 with (**B**) pABLAC1 and (**C**) pKCRI-28-1. Genes, open reading frames, iterons, IS, CR2, and p*dif* sites are shown as in Fig. 1 and 2. The *ser* gene is orange, and *sel1* is teal. The locations of Tn*7735*, Tn*6252*, ISAba33, and tISAba43 are indicated by a vertical bar. Regions of significant DNA identity are indicated by gray shading, with the percent identity also shown. Figures drawn to scale from GenBank accession numbers listed in Table 1 for pNL6-2 and pNL6-3 and GenBank accession numbers KY216144 for pRCH51-3, CP007713 for pABLAC1, and LR026972 for pKCRI-28-1.

The r3-T20 plasmids pNL6-3 and pRCH51-3 share a backbone made up of several segments with nucleotide identities ranging from as low as 95.47 to 99.58% (Fig. 3A), but there are several insertions or deletions, which differ between the two plasmids. A unique region includes almost all of the antibiotic resistance genes present in each plasmid; a 28,265 bp region in pNL6-3 is replaced by a 20,096 bp region that includes *oxa235*, *sul2*, and *floR* resistance genes in pRCH51-3 (Fig. 3A). No IS or p*dif* site could explain the replacement of this region. The backbone segment includes *repA* and genes that encode ParA and ParB partitioning proteins, *umuC* and *umuD*, which are involved in DNA repair after damage, as well as putative *mobA* and *mobC* genes (shown at the left end in Fig. 3A). The rest of the shared backbone includes *topA* encoding a DNA topoisomerase and a set of 10 genes previously predicted to be involved in conjugative transfer (41, 44–46) (blue genes, Fig. 3A) on the basis that they encode proteins that are distantly related to the Vir conjugative transfer proteins of the pTi plasmid (MPF_t_ system) (41).

### Conjugation of pNL6-3

As pNL6-3 appears to have a complete set of conjugative transfer genes, movement of pNL6-3 from the original strain into a new host was tested. Recipients were rifampicin-resistant mutants of ATCC17978 or the derivative ATCC17978-A, which lacks the large conjugative plasmid pAB3. pNL6-3 transferred at a frequency of 1.16 × 10^−4^ Km^R^Rif^R^ transconjugants/Nx^R^ donor into ATCC17978^rif^ or 4.20 × 10^−4^ Km^R^Rif^R^ transconjugants/Nx^R^ donor into ATCC17978^rif^-A, as an average of three independent replicates. Fifty transconjugants screened were all susceptible to nalidixic acid (ATCC17978^rif^ is Nx^S^). The presence of the r3-T20 plasmid in the recipient was confirmed by PCR screening of 10 transconjugants for the plasmid *repA* gene and for the *gtr100* gene, which is present in KL49 in NL6 but not in ATCC17978^rif^, confirming that they were not rifampicin-resistant mutants of the donor. The resistance profile of three representative transconjugants revealed they were resistant to kanamycin, neomycin, gentamicin, tobramycin, netilmicin, ceftazidime, cefotaxime, cefepime, and ceftriaxone (Table 2), which is consistent with the profile expected from the antibiotic resistance genes found in the plasmid. Trimethoprim resistance was not conferred by the plasmid, indicating it was not responsible for the unexplained trimethoprim resistance.

**TABLE 2:** Phenotypic antibiotic resistance profile of donor, recipients, and representative transconjugants

Isolate^*a*^	Annular radius of inhibition zone (mm)^*b*^*^,^*^*c*^												
	CAZ	CTX	FEP	CRO	Km	Nm	Gm	Tm	Ak	Net	Tp	Rif	Nx
NL6^*d*^	0	0	1	0	0	4.5	0	4.5	4	6	0.5	7	0
ATCC17978^rif*e*^	7	6	6	6	7.5	7	7.5	7	7	8.5	5	0	7
ATCC17978^rif^-A^*e*^	6.5	6	7	6	7	7	7	8	7	8	6	0	6.5
T/C 1 (ATCC17978^rif^)	1	0	1	0	0	4	0	5	5	4	6	0	7.5
T/C 2 (ATCC17978^rif^)	0	0	1	0	0	4	0	5	4.5	4.5	5	0	7.5
T/C 3 (ATCC17978^rif^)	0	0	1	0	0	4	0	4.5	4.5	4.5	6	0	7
T/C 1 (ATCC17978^rif^-A)	0	0	1	0	0	4	0	4	3.5	5	6	0	7
T/C 2 (ATCC17978^rif^-A)	1	0	1	0	0	4	0	4.5	4	4.5	5	0	7
T/C 3 (ATCC17978^rif^-A)	1	0	1	0	0	4	0	4.5	4.5	5	6	0	7.5

^*a*^ T/C—transconjugant.

^*b*^ Annular zone of inhibition of <6 mm indicates resistance.

^*c*^ CAZ, ceftazidime; CTX, cefotaxime; FEP, cefepime; CRO, ceftriaxone; Km, kanamycin; Nm, neomycin; Gm, gentamicin; Tm, tobramycin; Ak, amikacin; Net, netilmicin; Tp, trimethoprim; Rif, rifampicin; Nx, nalidixic acid.

^*d*^ Donor.

^*e*^ Recipient.

Previously, transfer by conjugation of pRCH51-3 into *A. baumannii* strain AB307_0294 was not detected (41). Here, pRCH51-3 was transferred into different recipients, ATCC17978^rif^ or ATCC17978^rif^-A, but at extremely low frequency, with a total of only seven or five Tic^R^Rif^R^ transconjugants obtained. PCR screening for the *repA* gene confirmed that the plasmid had transferred, and PCR screening for the *wzy* gene located in GI-2 in RCH51 (47) confirmed that they were not rifampicin-resistant mutants of the donor. The *virD4* gene, which encodes the coupling protein, has different lengths in pNL6-3 (2,871 bp; 956 aa) and pRCH51-3 (2,418 bp; 805 aa), though there are other differences in the DNA identity across the transfer region in these two plasmids (see Fig. 3A), which could contribute to the observed differences in conjugation frequency.

### pNL6-2

Plasmid pNL6-2 (15,038 bp; copy number 2.99) includes the *tet39 dif* module. This plasmid is an r3-T5 type with a 3.27 kb backbone C module that is identical to the backbone of the r3-T5 reference plasmid pABLAC1, which was found in LAC-4 (48). These plasmids are part of the recently defined Rep_3/OrfX plasmid group, all of which carry at least one *dif* module (49). The accessory region of pNL6-2 comprises four *dif* modules (Fig. 2B), two D modules (*tet39* is in a D module, and a second D module carries orf105), one C module containing the *higBA2* toxin-antitoxin gene pair and orf69, and a CD fusion module. The CD module includes three open reading frames that encode an N(4)/N(6)-adenine-specific DNA methyltransferase (IPR050953), a protein with a BsuBI/PstI restriction endonuclease domain (IPR009528), and a helicase and restriction-modification enzyme (IPR050742) (pale green arrows in Fig. 2B). This module is also interrupted by a copy of tISAba43, which generated an 8 bp TSD.

While pNL6-2 and pABLAC1 have an identical backbone, they only share two *dif* modules, the *higBA2*-orf69 and orf105 modules, which are adjacent to one another in both plasmids but in opposite orientations relative to the backbone module (Fig. 3B). Comparison of pNL6-2 with other previously described r3-T5 plasmids (48) revealed that the four *dif* modules present in pNL6-2 are also present in the r3-T5 + r3-T10 cointegrate plasmid pKCRI-28-1 (LR026972) (50). However, they have a different configuration in respect to both order and orientation relative to the backbone, indicating multiple recombination events have occurred (Fig. 3B).

### pNL6-1, a novel Rep_3 family plasmid

The smallest plasmid, pNL6-1 (2,471 bp), has a very high copy number of 27. It has two open reading frames (Fig. 2A) but does not carry any antibiotic resistance genes. It encodes a Rep_3 family replication initiation protein (IPR000525) but could not be typed using the *Acinetobacter* plasmid typing tool as the *repA* gene shares <75% aa identity with any known *Acinetobacter repA* gene encoding a Rep_3 family protein. This novel r3 type was assigned the number r3-T204 (M. Hamidian, personal communication). The second open reading frame encodes a hypothetical protein (106 aa), which has a winged HTH domain (IPR055245). There are four directly repeated copies of an iteron sequence located 45 bp upstream of *repA*. The first three are identical and 22 bp in length (5′- TTACCTTGCAATAAGACGACAT-3′), the fourth is 21 bp and missing the A at the 10th or 11th base of the iteron sequence. No related plasmids were identified in the GenBank non-redundant database (last searched 15 February 2026).

### Features of NL6 shared with LAC-4

Given that both the NL6 and LAC-4 chromosomes have Tn*6250* or Tn*6250*Δ in the same location, we also examined whether other features were shared by NL6 and LAC-4. The chromosomes differ in length (3,777,870 bp and 3,954,434 bp), and comparison of their overall chromosome structure revealed that a large portion of the chromosome (~1.2 Mb) is inverted in NL6 relative to LAC-4. However, this inversion occurred within regions unique to NL6 or LAC-4, and its cause was not pursued further.

In addition to the copies of IS*1006* and ISAba1 in Tn*6250*, 16 of the IS in the NL6 chromosome are present in the same position in LAC-4 (shared IS are indicated by a * in Fig. 1A). This included copies of ISAba26, ISAba13, and ISAba1. LAC-4 has additional copies of ISAba1, ISAba13, ISAba26, and >10 copies of both ISAba25 and ISAba125, two IS which are not present in NL6 (Table 4). NL6 does not include a copy of Tn*6252*, found in the LAC-4 chromosome, which carries the carbapenem resistance gene *oxa235* (24).

A copper resistance region first identified in LAC-4 is located between two copies of ISAba26 and called GI2 in reference 24 (see Fig. 3 in [24] for the structure of the region) is also present in NL6 (Fig. 1A). However, a TSD was not present around this region, indicating it was not likely to have been mobilized by the two copies of ISAba26, which we found are each surrounded by a TSD. Comparison of this region with the chromosomes of other ST10 isolates that lack the copper resistance genes revealed an integrative element (IE), here designated IECu^R^, of 29.6 Kbp, excluding the ISAba26 copies, as well as an ISAba25 copy in LAC-4 and a tISAba43 copy in NL6 (3,223,292–3,258,271 in the NL6 chromosome; accession number CM146746).

Several other potential genomic islands and transposons were identified previously in the complete genome of LAC-4 (24). Two of them, called GI11 and GI4, are present in NL6, though in LAC-4, there are additional IS (ISAba25 or ISAba26) inserted into these islands (see [24] for additional information about the structure of these genomic islands). The proposed GI3 in LAC-4 is surrounded by ISAba25s and includes the *adeI*, *adeJ*, and *adeK* genes that are widespread in *A. baumannii* chromosomes. This region is present in NL6, but the ISAba25s are not. Several additional regions in LAC-4 were described as encoding phage proteins (including GI5, GI6, GI7, GI8, GI9, and GI10). However, only GI8 was present (92% coverage) in NL6, and this region was not identified as a putative prophage by PHASTEST. In addition, in LAC-4, there is a prophage related to, and in the same location as, prophage one in NL6.

### Relationship to other complete ST10 genomes

**Fig 4:**
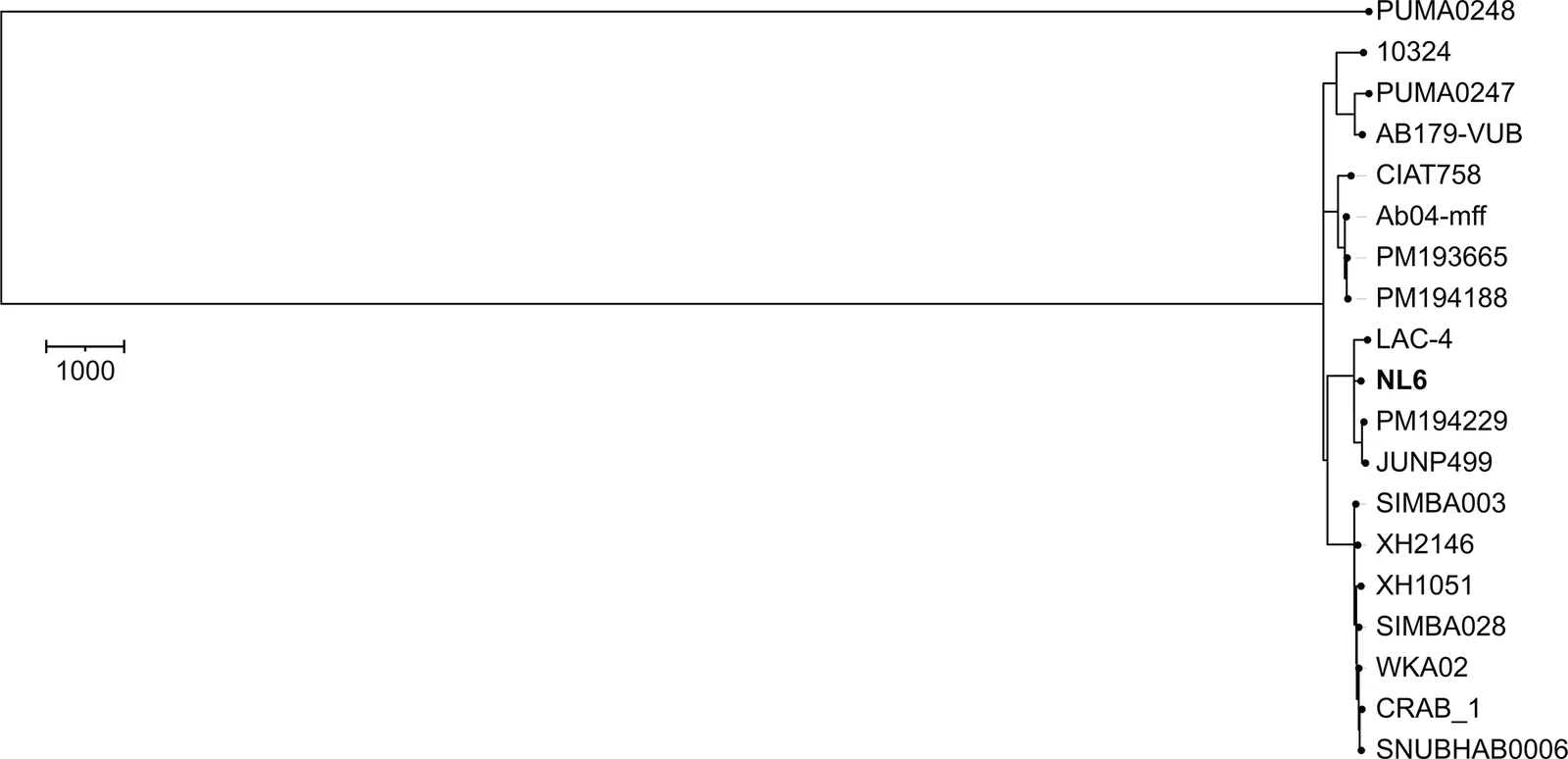
Core SNP phylogeny of ST10 isolates, which have complete genomes. The maximum-likelihood tree was inferred using RAxML (V8) with the GAMMA model based on core SNPs aligned against the reference genome LAC-4 (CP007712). Recombinant regions were removed prior to tree building using Gubbins (v2.1.025). Scale bar represents 1,000 core SNP differences.

A total of 20 complete ST10 genomes were found in GenBank (downloaded 16 July 2025, Table 3), but two are duplicates of LAC-4 and Ab04. Most were isolated in Asia. Only 13 of the 20 included KL49; two included KL9; and 5 KL types occurred only once. Examination of the mobile elements in the chromosomes revealed that only LAC-4, NL6, and two others carried both Tn*6250* and IECu^R^ (Table 4). These four isolates were also grouped together in a phylogeny (Fig. 4). However, a second group made up of Ab04 and two others carried only Tn*6250*. All but one of the chromosomes carried IS and the IS-free isolate did not group with the others (Fig. 4), suggesting it may not be of the main ST10 lineage. Examination of the antibiotic resistance genes present and their location on a plasmid or the chromosome revealed a diverse collection of resistance genes and plasmids (Table 5). Ten of the additional ST10 genomes included *oxa23*, indicative of carbapenem resistance, and five of these also included *armA* (amikacin, gentamicin, netilmicin, tobramycin, and kanamycin resistance) usually in a large conjugative plasmid (LCP). The clustering of resistance genes that co-occur was not pursued.

**TABLE 3:** Complete sequences of ST10 *A. baumannii* isolates in GenBank

Strain	KL	OCL	Year^*a*^	Country^*a*^	Isolation source^*b*^	Accession	Reference^*a*^
LAC-4^*c*^	49	2	1997	USA	Clinical	GCA_000786735	(24)
LAC4^*c*^	49	2	1997	USA	Clinical	GCA_001908295	–
PM194229	49	2	2019	India	BAL	GCA_012935125	(29)
JUNP499	49	2	2019	Nepal	Human	GCA_041154835	(51)
Ab04-mff^*d*^	49	2	2012	Canada^*e*^	Blood	GCA_001077655	(12)
HvAb04^*d*^	49	2	–^*a*^	–	Human	GCA_042142205	–
PM193665	9	2	2019	India	Pus	GCA_012935085	(30)
PM194188	9	2	2019	India	BAL	GCA_012935105	(30)
XH2146	49	2	2021	China	CAP	GCA_040357515	(19)
SIMBA003	49	2	2006	Singapore	Human	GCA_040822125	–
SIMBA028	49	2	2008	Singapore	Human	GCA_040822395	–
XH1051	49	2	2010	China	Sputum	GCA_043040895	(52)
SNUBHAB0006	49	2	2018	South Korea	Bronchial fluid	GCA_045278625	–
CRAB_1	49	2	2018	South Korea	Blood	GCA_045037785	–
WKA02	49	2	2013	South Korea	Sputum	GCA_002082805	(31)
AB179-VUB	58	2	2017	Belgium	Sputum	GCA_022459075	(32)
10324	77	2	2012	Mexico	Bronchial fluid	GCA_003522705	(53)
CIAT758	108	2	2018	India	Blood	GCA_004758865	(54)
PUMA0247	125	2	2019	–	–	GCA_036701095	–
PUMA0248	136	2	2019	–	–	GCA_036689575	–

^*a*^ “–”, data not available.

^*b*^ BAL—bronchoalveolar lavage; CAP—community-acquired pneumonia.

^*c*^ Two versions of the complete genome for LAC-4 are available in GenBank; the original entry is available under assembly accession number GCA_00786735.

^*d*^ Two versions of the complete genome for Ab04 are available in GenBank; the original entry is available under assembly accession number GCA_001077655.

^*e*^ Ab04 is from a hospital outbreak in Canada. The index patient for this outbreak had a recent history of hospitalization in India.

**TABLE 4:** Insertions in ST10 chromosomes

Strain	KL	OCL	Cu^R^ GI	Tn6250^*a*^	ISAba1	ISAba13	ISAba14	ISAba25	ISAba26	ISAba33	tISAba43	ISAba125
LAC-4	49	2	+	+	19	22	–^*f*^	12	14	–	–	14
LAC4	49	2	+	+	20	24	–	15	14	–	–	16
NL6	49	2	+	+^*b*^	12	7	1	–	12	4	6	–
PM194229	49	2	+	+	8	10^*c*^	–	–	15	–	–	–
JUNP499	49	2	+	+^*d*^	16	12	–	–	13	–	–	–
AB04-mff	49	2	–	+	5	4	4	–	10	–	–	12
HvAb04	49	2	–	+	5	4	5	–	10	–	–	12
PM193665	9	2	–	+	8	7	3	–	10	–	–	12
PM194188	9	2	–	+	9	8	3	–	10	–	–	17
XH2146	49	2	–	–	1	–	–	–	10^*e*^	–	–	–
SIMBA003	49	2	–	–	1	–	–	–	6^*e*^	–	–	–
SIMBA028	49	2	–	–	1	–	–	–	6^*e*^	–	–	–
XH1051	49	2	–	–	6	–	–	–	6^*e*^	–	–	–
SNUBHAB0006	49	2	–	–	18	–	–	–	7		–	–
CRAB_1	49	2	–	–	14	–	–	–	6	–	–	–
WKA02	49	2	–	–	4	–	–	–	8	–	–	–
AB179-VUB	58	2	–	–	2	–	–	–	20	–	–	–
10,324	77	2	–	–	–	–	–	–	12	–	–	–
CIAT758	108	2	–	–	4	–	1	–	9	–	–	1
PUMA0247	125	2	–	–	–	1	–	–	16	–	–	–
PUMA0248	136	2	–	–	–	–	–	–	–	–	–	–

^*a*^ Tn*6250* includes a copy of IS*1006*.

^*b*^ Has Tn*6250*Δ, a deletion derivative of Tn*6250*.

^*c*^ One copy of ISAba13 is interrupted by a copy of ISAba34.

^*d*^ Additional insertion of a Tn*1548*-like region within Tn*6250* that includes the rest of resistance genes in JUNP499, except *oxa23*.

^*e*^ An additional partial copy of ISAba26 is present in the chromosome.

^*f*^ "–” indicates no copies are present in the chromosome.

**TABLE 5:** Plasmids and antibiotic resistance genes in ST10 isolates

Strain	Chromosome/plasmid	Accession	Plasmid type^*a*^	Antibiotic resistance genes
LAC-4^*c*^	Chromosome	CP007712		*strAB*, *sul2*, *oxa235*
	pABLAC1	CP007713	r3-T5	–^*b*^
	pABLAC2	CP007714	pRAY*	*aadB*
LAC4^*c*^	Chromosome	CP018677		*strAB*, *sul2*, *oxa235* (2), *aphA6* (2)
	pALAC4	CP018678	r3-T5	–
	pALAC4-2	CP018679	pRAY*	*aadB*
PM194229	Chromosome	CP050432		*strAB*, *sul2*
	pPM194229_1	CP050433	LCP	*strAB*, *sul2* (2), *tet*(B) *msrE*, *mphE*, *armA*, *bla*_PER-7_, *arr2*, *cmlA1*, *sul1* (2)
	pPM194229_2	CP050434	r3-T2	–
	pPM194229_3	CP050435	r3-T5	–
JUNP499	Chromosome	AP031582		*strAB* (2), *sul2* (3), *oxa23* (2) *bla*_NDM-1_ *msrE*, *mphE*, *armA*, *bla*_PER-7_, *arr2*, *cmlA1*, *sul1* (2)
Ab04-mff^*d*^	Chromosome	CP012006		*strAB*, *sul2*, *oxa23*, *aphA6*
	pAB04-1	CP012007	LCP	*strAB*, *sul2* (2), *tet*(B) *msrE*, *mphE*, *armA*, *bla*_PER-7_, *arr2*, *cmlA1*, *sul1* (2)
	pAB04-2	CP012008	rP-T1	*oxa23*
HvAb04^*d*^	Chromosome	CP169805		*strAB*, *sul2*, *oxa23*, *aphA6*
	Plasmid 1	CP169806	LCP	*strAB*, *sul2* (2), *tet*(B) *msrE*, *mphE*, *armA*, *bla*_PER-7_, *arr2*, *cmlA1*, *sul1* (2)
	Plasmid 2	CP169807	r3-T11	–
	Plasmid 3	CP169808	rP-T1	*oxa23*
PM193665	Chromosome	CP050415		*strAB*, *sul2, oxa23, aphA6*
	pPM193665_1	CP050416	LCP	*strAB*, *sul2* (2), *tet*(B), *bla*_NDM-1_ *msrE*, *mphE*, *armA*, *arr2*, *cmlA1*, *sul1*
	pPM193665_2	CP050417	N/A^*e*^	–
	pPM193665_3	CP050418	r3-T11	–
	pPM193665_4	CP050419	r3-T1	–
	pPM193665_5	CP050420	r3-T30	–
PM194188	Chromosome	CP050425		*strAB*, *sul2, oxa23*
	pPM194122_1	CP050426	LCP	*strAB*, *sul2* (2), *tet*(B), *bla*_NDM-1_ *msrE*, *mphE*, *armA*, *arr2*, *cmlA1*, *sul1*
	pPM194122_2	CP050427	r3-T11	–
	pPM194122_3	CP050428	r3-T17	–
	pPM194122_4	CP050429	r3-T1	–
	pPM194122_5	CP050430	r3-T30	–
	pPM194122_6	CP050431	N/A^*f*^	
XH2146	Chromosome	CP128372		–
	pXH2146-1	CP128373	r3-T3	*sul2*, *tet*(B)
	pXH2146-2	CP128374	r3-T5	–
SIMBA003	Chromosome	CP161986	–	*–*
	pSIMBA003_1	CP161987	r3-T3	*sul2*, *tet*(B)
	pSIMBA003_2	CP161988	r3-T5	–
SIMBA028	Chromosome	CP161989		
	pSIMBA028_1	CP161990	r3-T3	*sul2*, *tet*(B)
	pSIMBA028_2	CP161991	r3-T5	–
XH1051	Chromosome	CP134548		*oxa23* (2)
SNUBHAB0006	Chromosome	CP137075		*sul2*, *oxa23* (3)
	pA0006	CP137076	rP-T1	–
CRAB_1	Chromosome	CP173428		*sul2*, *oxa23* (2), *tet*(B)
	Plasmid 1	CP173429	rP-T1	–
WKA02	Chromosome	CP020598		*sul2*, *oxa23*, *tet*(B)
AB179-VUB	Chromosome	CP091360		–
10324	Chromosome	CP023022		
	pAba10324a	CP023023	r3-T18	–
	pAba10324b	CP023024	r3-T5	–
	pAba10324c	CP023025	r3-T3	*sul2*, *tet*(B)
CIAT758	Chromosome	CP038500		*oxa23*
	Plasmid 1	CP038501	r3-T21	*tet*39, *aacC2*, *msrE*, *mphE*, *oxa58*
	Plasmid 2	CP038502	ND^*g*^	*bla*_PER-7_, *sul1*, *aphA6*
	Plasmid 3	CP038503	ND^*g*^	
PUMA0248	Chromosome	CP144757		–
PUMA0247	Chromosome	CP144907		–
	pPUMA0247_1	CP144908	r3-T5	–
	pPUMA0247_2	CP144909	r3-new^*h*^	

^*a*^ Plasmid *rep* genes typed using *Acinetobacter* plasmid typing scheme (38). ND—not determined, N/A—not applicable. LCP—large conjugative plasmid of the pAB3/pA297-3 type. LCP and pRAY*-type plasmids lack an identifiable *repA* gene and are identified by comparison to pAB3(CP012005)/pA297-3(KU744946) and pRAY*(JQ904627), respectively.

^*b*^ “–”, indicates no antibiotic resistance genes are present.

^*c*^ Two versions of the complete genome for LAC-4 are available in GenBank; the original entry is available under assembly accession number GCA_00786735.

^*d*^ Two versions of the complete genome for Ab04 are available in GenBank; the original entry is available under assembly accession number GCA_001077655.

^*e*^ The sequence appears to be a circularized phage and not a plasmid. This sequence is also present in the chromosome of strain PM194188 (CP050425).

^*f*^ This sequence does not appear to be a plasmid. It consists only of 23s rRNA and likely results from misassembly of the genome.

^*g*^ The plasmid could not be typed using the *Acinetobacter* typing scheme.

^*h*^ This is a novel *repA* type. The gene sequence shares 94.08% DNA identity with the *repA* of the r3-T153 type.

### Conclusions

NL6 is typical of the ST10 isolates for which complete genome sequences were available in that most of its resistance genes were brought in by plasmids. The NL6 plasmids and the resistance genes they carry were not shared by other ST10 genomes. However, acquired features in the chromosome indicated an evolutionary history shared with a few other ST10 chromosomes, and the phylogeny supported that grouping.

## MATERIALS AND METHODS

### Bacterial isolate and antibiotic sensitivity testing

*A. baumannii* NL6 was kindly supplied by Prof. Stephen Baker. NL6 was screened for resistance to 29 antibiotics using a calibrated disc diffusion assay as described previously (13).

### Genome sequencing

Whole cell DNA was extracted as described previously (55) and sent for long read sequencing using Oxford Nanopore technology at Plasmidsaurus (San Francisco, United States). A total of 17,187 reads were obtained (average length 8,345 bp; 37.3× coverage). The long reads (SRA accession number SRR37355546) were combined with available 150 bp paired-end Illumina MiSeq reads (48-fold coverage; SRA accession number SRR33920457) using Hybracter (v0.11.2) (56) with default parameters to generate a hybrid assembly of the NL6 genome.

### Bioinformatic analysis

Antibiotic resistance genes present in NL6 and the *oxaAb* allele were determined using National Center for Biotechnology Information (NCBI) AMRFinder+. Plasmids were typed using the *Acinetobacter* plasmid typing tool (38). Plasmid copy number was estimated using the average Illumina read depth for the plasmid relative to the average Illumina read depth of the seven MLST^IP^ alleles. Insertion sequences (IS) were identified using ISFinder (https://tncentral.ncc.unesp.br/ISfinder/). The p*dif* sites and *dif* modules in plasmids were identified manually by first searching for the more conserved XerD binding site then looking 6 bp away for a XerC binding site and annotated as described previously (39, 57). The protein coding regions, rRNA, and tRNA genes in the NL6 genome were automatically annotated using Prokka (v 1.12). Annotations of regions of interest, including IS, KL, and OCL, plasmids, regions containing antibiotic resistance genes, *dif* modules, and transposons were enhanced manually using BLAST searches of the GenBank non-redundant nucleotide database or in-house collections of known resistance gene associated regions. Predicted phage regions were identified using PHASTEST (https://phastest.ca/). Core SNP phylogeny of available ST10 complete genomes (downloaded 16 July 2025) was performed as described previously (8) using LAC-4 as the reference genome.

### Conjugation assays

Conjugation of pNL6-3 from NL6 (nalidixic acid resistant) or pRCH51-3 from RCH51 into rifampicin-resistant mutants of *A. baumannii* strain ATCC 17978 (10) or *A. baumannii* ATCC17978-A, which lacks pAB3 (14), was performed as described previously (14). Briefly, equal volumes of stationary-phase overnight cultures of donor and recipient cells were mixed on an LB agar plate and grown overnight at 37°C. Cells were harvested in 0.9% (w/v) saline, and transconjugants were selected by plating on L Agar plates containing rifampicin (100 mg/L) and kanamycin (20 mg/L) for pNL6-3 or rifampicin (100 mg/L) and ticarcillin (100 mg/L) for pRCH51-3. Conjugation frequencies were calculated as the number of transconjugants/donor as an average of three independent experiments.

Putative transconjugants containing pNL6-3 were purified and screened for growth on L agar containing nalidixic acid, to which the donor was resistant and the recipient was susceptible, to confirm that they were not rifampicin resistant mutants of the donor. PCR was performed as described previously (58) to screen for the presence of the plasmid in transconjugants. Primers RH3378 (5′-TTGACCTCCCCAATGGCTAG-3′) and RH3379 (5′-CCCTCGTTTTGCCTGTAACC-3′) were used to detect the r3-T20 *repA* gene. PCRs to amplify the *gtr100* gene in KL49 of NL6 using primers RH3384 (5′-TTTGACAAAACAGTTAACCACCT-3′) and RH3385 (5′-ACGAGCAACAGTACAATTTCCT-3′) or the *wzy* gene in RCH51 using primers RH3380 (5′-ACCGCCATTGTAGAACAGGA-3′) and RH3381 (5′-TTTCGCGGTTTCTATGCCAA-3′) were performed to confirm that transconjugants were not rifampicin resistant mutants of the donor. Three purified transconjugants containing pNL6-3 were screened for resistance to ceftazidime, cefotaxime, cefepime, ceftriaxone, kanamycin, neomycin, gentamicin, tobramycin, amikacin, netilmicin, trimethoprim, rifampicin, and nalidixic acid using the calibrated disc diffusion assay as described for the NL6 strain.

## Data Availability

The sequence data for NL6 have been deposited under BioProject number PRJNA893697 and BioSample Number SAMN48993107. Accession numbers for the complete genome of NL6 are GCA_050971825.2 for the assembly, CM146746 for the chromosome, JBPCCI020000002 for pNL6-1, JBPCCI020000003 for pNL6-2, and JBPCCI020000004 for pNL6-3.
